# Examining the use and weight transfer of four custom‐made partial hindlimb exoprostheses for a dog

**DOI:** 10.1002/vro2.70039

**Published:** 2026-07-06

**Authors:** Aljaž Muršec, Monika Pavlović, Jurij Žel, Vladimira Erjavec

**Affiliations:** ^1^ Faculty of Health Sciences University of Ljubljana Ljubljana Slovenia; ^2^ Metropolia University of Applied Sciences Helsinki Finland; ^3^ Veterinary Faculty University of Ljubljana Ljubljana Slovenia

**Keywords:** amputation, canine, dog, force plate, limb exoprosthesis

## Abstract

**Background:**

Limb exoprosthetics in veterinary medicine are less developed than in human medicine. This case study evaluates four custom‐made hindlimb exoprostheses for a 6‐year‐old dog following metatarsal amputation due to a train accident.

**Methods:**

After wound healing, four exoprostheses—differing in measurement methods and materials—were tested over 4 weeks, each for 1 week. Assessment tools included force plate measurements, accelerometer‐based activity tracking and owner feedback. Force plates evaluated load distribution across the exoprosthetic limb, healthy hindlimb and forelimbs.

**Results:**

The second exoprosthesis, made of polypropylene using a double‐mould method, had the highest user acceptance and activity levels. The PitPat tracker recorded an average of 41.4 min of walking per day, with 6.97% of bodyweight transferred to the exoprosthesis. The third and fourth exoprostheses caused skin abrasions and were discontinued after 3 days.

**Conclusion:**

Well‐designed exoprosthetics can improve limb use in dogs with partial amputation. Force plates, activity trackers and owner feedback are useful outcome measures for evaluating exoprosthesis performance and enhancing quality of life.

## INTRODUCTION

Exoprosthetic devices improve the mobility and activity of animals with amputated limbs, enabling them to maintain an active lifestyle and reduce the risk of secondary musculoskeletal problems due to biomechanical changes.^1^, ^2^ Exoprostheses are custom‐made medical devices that replace the function of the missing limb segment and provide mechanical support during standing and locomotion.^3^ They consist of three basic parts: a socket, a part for adjustment of the height of the missing limb and a part with surface contact. Other specific components can be added if required by the individual case. The socket is shaped to fit the stump and is attached with straps that secure the exoprosthesis to the stump so that it does not move during activity. Its outer part is made of a rigid or semi‐rigid material, while the interior, in contact with the skin, is composed of foam or silicone to prevent stump trauma. Shank or pylons are the parts of the exoprosthesis that are responsible for height compensation and adequate force transfer. The distal part of the exoprosthesis is on the floor and provides sufficient support and prevents slipping on various surfaces.^2^, ^4^ In humans, and also in veterinary medicine, advances in materials and technology, such as carbon fibre and 3D scanning and printing with different materials such as polypropylene, polyethylene terephthalate glycol‐modified, thermoplastic polyurethane, acrylonitrile butadiene styrene and polylactic acid, have improved prosthetic functionality, weight and adaptability.^5^, ^6^, ^7^, ^8^

Partial limb amputations in dogs may be performed due to trauma, neoplasia, or severe infection.^6^, ^9^ Although limb‐sparing approaches preserve part of the limb, partially amputated limbs often exhibit compromised function, altered gait patterns and reduced load‐bearing capacity. Over time, inadequate limb use may contribute to secondary musculoskeletal changes, discomfort and reduced quality of life.^10^, ^11^ Exoprostheses have been introduced as a means of improving limb function in animals with partial amputations by providing external support, enhancing stability and facilitating more symmetrical limb use.^12^

While the clinical benefits of animal exoprostheses have been increasingly reported, their application is not yet routine, and objective data evaluating functional limb use in animals fitted with exoprostheses remain limited.^12^, ^13^, ^14^ In particular, little is known about how different exoprosthetic designs influence limb function in dogs with partial amputations.^15^, ^16^ Assessment of exoprosthetic effectiveness in animals is challenging due to the inability to obtain direct user feedback.^17^, ^18^ Consequently, evaluation relies on objective measurements of limb use and movement, combined with behavioral observation and owner‐reported outcomes.^19^, ^20^ Although several quantitative methods are well established in human exoprosthetic research, their application in veterinary patients is still emerging.^21^, ^22^

The aim of this study was to evaluate short‐term prosthetic use and limb function in a dog with a partial hindlimb amputation fitted sequentially with four different manufactured exoprostheses. Limb function was assessed using force plate analysis, an accelerometer‐based activity tracker (PitPat), and an owner questionnaire, in order to determine which exoprosthesis provided the most suitable functional outcome for this dog.

## MATERIALS AND METHODS

Before the study, the dog's owner was informed about the procedures and signed an informed consent form. The protocol of the patient treatment was approved by the Ethical Committee of the Veterinary Faculty, University of Ljubljana.

### Patient background

**FIGURE 1:**
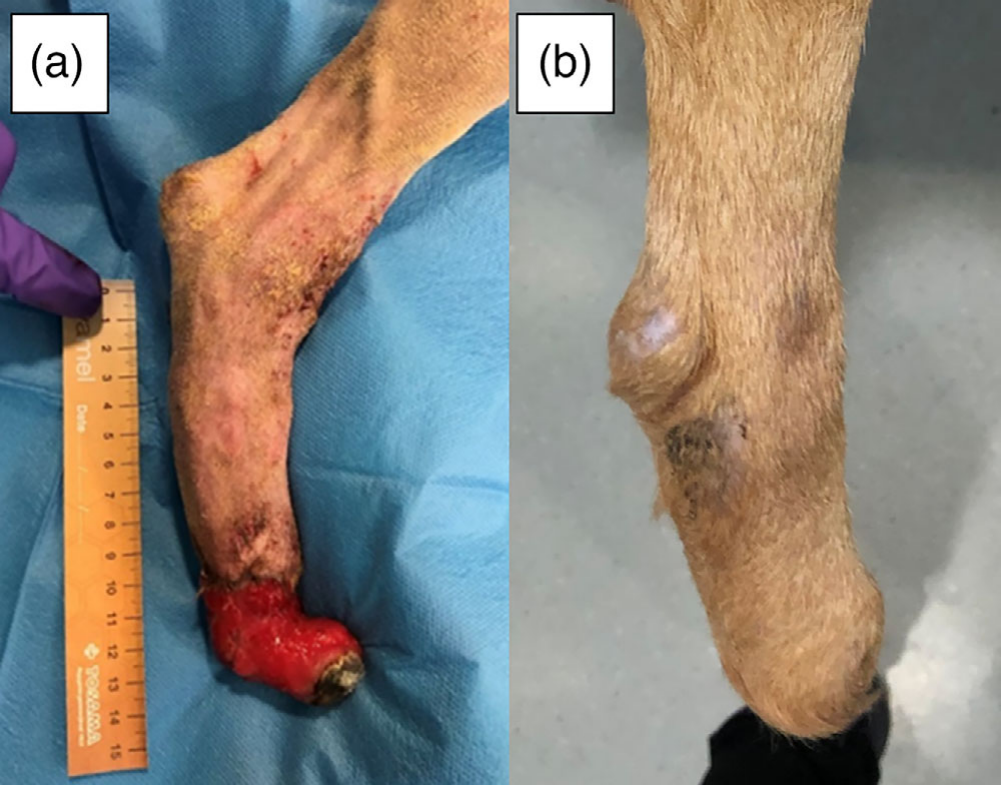
(a) Stump in the metatarsal region prior to amputation, resulting from trauma and unsuccessful wound healing. (b) Stump following amputation and surgical wound healing, at the time of exoprosthesis fitting.

A 6‐year‐old short‐haired, mixed‐breed dog, weighing 27.5 kg, had suffered a traumatic injury to the distal right hindlimb in a train accident 1 year prior (Figure 1).

Initially, the owner declined limb amputation, which led to the conservative treatment approach. However, this did not result in complete wound healing and satisfactory limb bearing. Only the third phalanx remained intact, while other parts of the distal limb were partially or completely removed. Necrotic areas appeared in the distal part of the wound, requiring regular debridement. Despite these efforts, the wound remained non‐healing, requiring bandaging twice weekly to manage extensive exudate and tissue maceration.

### Simple therapeutic exoprosthesis

To alleviate discomfort, a custom‐made simple exoprosthesis was initially applied, although clinical improvement was limited. In the fabrication of the exoprosthesis, 4 mm polypropylene was utilised as the primary material. The polypropylene was thermally processed in an infrared oven at 170°C. Upon reaching its forming tempaerature, the polypropylene was vacuum‐formed over the plaster model representing a replica of the limb to ensure precise adherence to its contours. Subsequently, the polypropylene was trimmed from the model, and the edges were sanded. A 2 mm layer of ethylene vinyl acetate foam was then applied for additional cushioning. The foam was heated to 120°C and moulded inside the socket. The distal components of the exoprosthesis were similarly moulded using the same material and heating technique. Finally, four Velcro straps were affixed to secure the exoprosthesis in place.

### Other interventions

Due to lack of clinical improvement, an amputation was performed at the level of the proximal third of the metatarsals, resulting in a 5 cm shortening of the hindlimb and facilitating future exoprosthetic fitting. Following the surgery, a modified version of the previously used exoprosthesis was employed until complete healing was achieved. The exoprosthesis was expanded on the distal part by heating the polypropylene material, as there was oedema due to the surgical procedure.

One month after the amputation, when the scar had partially formed and the oedema was no longer present, circumference measurements were taken at 2 cm from the distal end of the stump to the midpoint of the tibia. To determine the exoprosthesis length, measurements included the stump length (from the distal end to the tarsal joint and from the tarsal joint to the middle of the tibia) and the healthy hindlimb length in a standing position from the floor to the tarsal joint.

Apart from the amputation, the dog had no other known acute or chronic diseases except for contact dermatitis on the right front paw, which was occasionally protected with a sock.

The joint range of motion (ROM) for both the healthy and amputated hindlimbs was assessed using a goniometer, following the method described by Jaegger et al.^23^ Goniometer measurements quantify the angle through which a joint can move, providing an objective measure of its ROM. By aligning the device with the bones forming the joint, clinicians can assess flexibility, detect limitations and monitor changes over time (e.g., before and after interventions such as an exoprosthesis). These measurements were conducted before applying the first exoprosthesis and after testing the fourth exoprosthesis.

**FIGURE 2:**
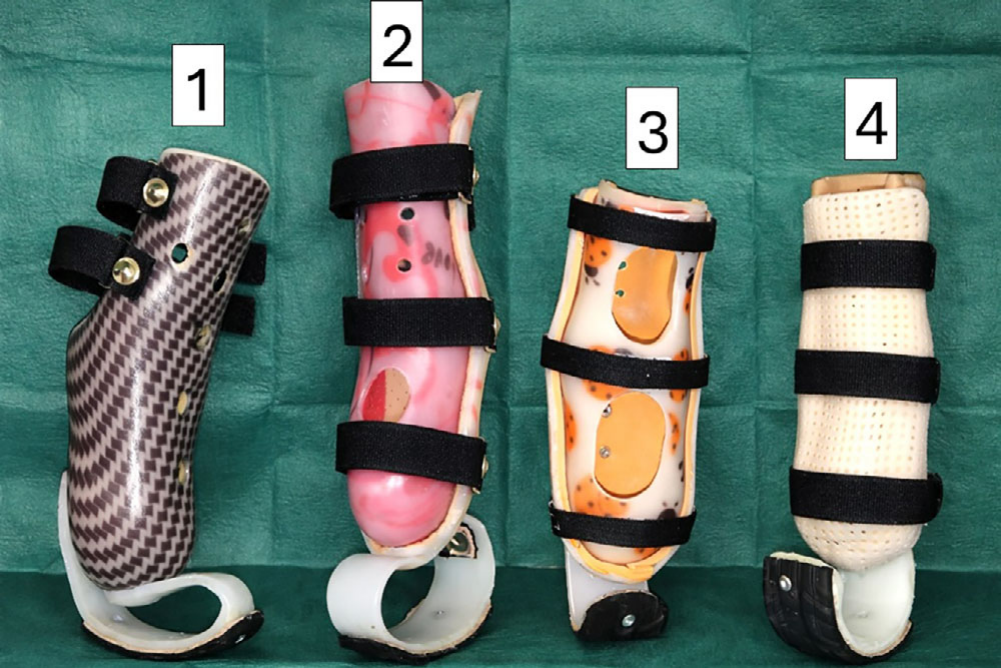
Loading and unloading regions on stump. Green areas indicate regions suitable for load bearing, where pressure can be safely distributed through the exoprosthesis. Red areas represent unloading zones corresponding to bony prominences or pressure‐sensitive regions, where direct load application should be minimised to reduce the risk of discomfort or tissue damage.

Two methods were used to create a negative mould of the stump: plaster bandages and alginate (a dental impression material). In the plaster bandage method, the stump was wrapped in several layers of plaster bandages. Once the material had hardened, the cast was carefully removed using plaster shears, and the incision was closed with an additional layer of bandage to maintain the integrity of the shape. Liquid plaster was then poured into the resulting shell. After the plaster had fully set, the bandages were removed, yielding a solid model of the stump. In the second method, based on the use of alginate, the stump was placed into a container filled with liquid alginate. Once the alginate had solidified, a negative mould of the limb was obtained. Liquid plaster was then poured into this mould, and after hardening, it formed an accurate model of the stump. These moulds served as the basis for creating plaster models of the stump, enabling the fabrication of multiple copies. Four exoprostheses were made (Figure 2) using various thermoplastic materials and distal components (Table 1).

**TABLE 1:** Manufacturing methods and properties of the manufactured prostheses.

Exoprosthesis	Measurement method	Materials	Method of manufacture	Distal part	Weight (g)
1	Plaster bandage	Polypropylene 4 mm	Single mould^a^	Rounded	89
2	Plaster bandage	Polypropylene 4 mm	Double mould^b^	Rounded	96
3	Alginate	Polypropylene 4 mm	Double mould^b^	Semicircle	74
4	Alginate	Low‐temperature thermoplastic 2 mm, distal part 4 mm	Double mould^b^	Semicircle	62

^a^ An exoprosthesis made from one piece of material, covering the stump on one side.

^b^ An exoprosthesis made from two pieces of material, surrounding the stump.

**FIGURE 3:**
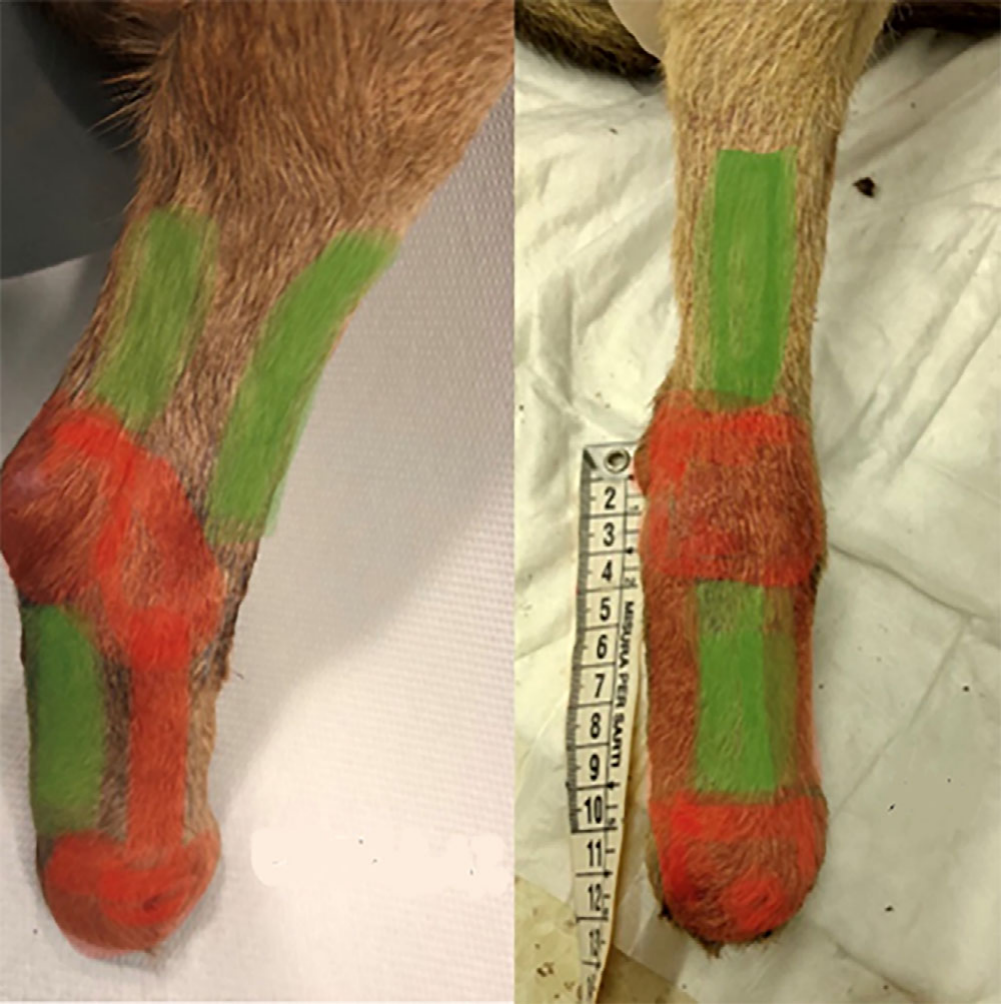
The exoprostheses (labelled 1–4) differ in design, materials and fixation mechanisms, including variations in structure, padding, ventilation and strap configuration. All prostheses were designed for the same dog and are shown without being worn to allow clear comparison of their structural and functional characteristics.

When designing the exoprosthesis socket, careful consideration was given to the relief and loading points, as these areas bear significant weight. In our case, the loading (green) and unloading (red) points were distributed as shown in Figure 3.

Points on the limb were assessed by clinical examination. The loading points (green) are located in areas of soft tissue (muscle and fat tissue), where there is less risk of skin injury from pressure. The unloading points (red) are located in areas with bone points and scar tissue, where the risk of skin damage is higher. The Achilles tendon was also considered an unloading point due to its vulnerability to pressure‐related injuries. The unloading points (red) were created by adding 4 mm of plaster to the model, while the loading points (green) were formed by removing 4 mm of plaster through grinding.

The same method and materials were used to fabricate exoprostheses 1, 2 and 3 as described for the orthosis. In contrast, exoprosthesis 4 was made using a low‐temperature plastic, which was heated in a water bath at 70°C and moulded over the model. The same material and heating methods were used for the distal part.

### Data collection

Weight distribution across four custom‐made hindlimb exoprostheses and the remaining limbs was analysed using a force plate, a device that measures ground reaction forces (GRFs) exerted by the limbs during stance, to determine which exoprosthesis was most suitable for the dog. Owner observations regarding the dog's behaviour with each exoprosthesis were also considered to identify the optimal design. Testing of the exoprosthesis began 2 months after the amputation. During this period, scar maturation and bone healing caused changes in the shape and dimensions of the stump. To address this change, adjustments were made using socks and foam of different thicknesses to ensure a proper fit between an exoprosthesis and the stump. As in humans, it is recommended that animals receive a new exoprosthesis once the stump is fully formed.^24^ Therefore, a new model and one exoprosthesis were made after the measurements have been taken and the stump has been fully shaped. Each exoprosthesis was tested for 1 week, during which its performance was evaluated. If the dog exhibited signs of pain or any injury to the stump was observed, testing of the particular exoprosthesis was immediately discontinued. During testing, the dog's daily activity was measured using the accelerometer‐based PitPat tracker, worn on its collar, 24 h a day. Data were collected with the PitPat application (PitPat HQ), which ensured monitoring of various forms of physical activity (walking, running and playing) and resting periods of the animal. Additionally, after every new exoprosthesis was fitted, the dog's owner completed a weekly paper‐based questionnaire during the force plate measurement sessions to assess their satisfaction and observe changes in the dog's activity and habits. The questionnaire evaluated the owner's perception of the dog's motor skills, habituation to the exoprosthesis and placement of an exoprosthesis. Responses were rated on a scale from 1 to 5, with 5 being the highest possible score for each question. The questionnaire was divided into two parts.

In the first part, five motor skills were assessed: walking, trotting, cantering, explosive acceleration and sitting on command. Each skill was scored individually on a scale of 1‒5, with a maximum score of 25 points.

The second part consisted of five questions related to the dog's adaptation to the exoprosthesis, its motor skills before and during the use, the exoprosthesis’ fit, and the owner's satisfaction with the exoprosthesis. A maximum score of 25 points was also awarded for this section. The highest possible score for the entire questionnaire was 50 points.

**FIGURE 4:**
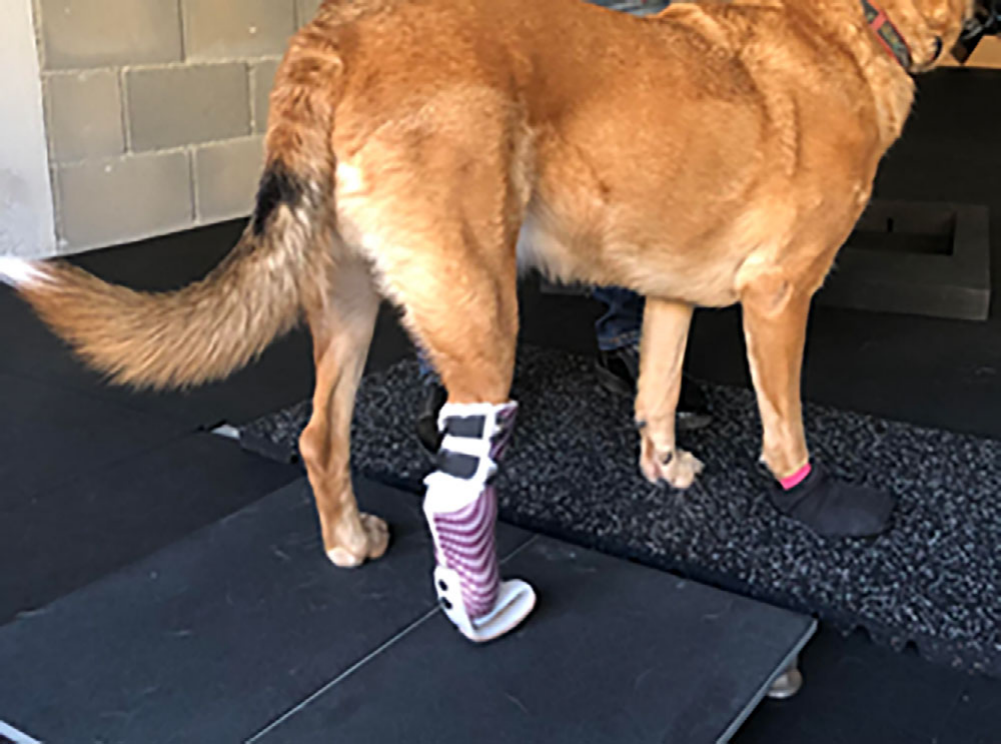
Experimental setup for kinetic data collection using a force plate. The dog is shown standing with the tested hindlimb fitted with the exoprosthesis placed on the force plate, while the contralateral healthy hindlimb is also positioned on the plate. The two forelimbs are supported on an elevated platform to ensure they are at the same height as the hindlimbs on the force plate, allowing proper weight distribution and accurate force measurements during static trials.

The mechanical load on each exoprosthesis was measured using a double force plate (ForceDecks Model FLite; Vald Performance). The force plates were leveled to the surrounding floor to ensure accurate measurements, preventing uneven weight distribution. During testing, the dog stood independently on the force plates with both hindlimbs—one fitted with the exoprosthesis and the other intact—while restrained with a loose leash (Figure 4).

The measurements were carried out statically while the dog was in a standing position. Measurements with inadequate positioning of the dog on the force plates were excluded. For each exoprosthesis, five measurements were taken after 1 week of use, with a 3‐min break between each measurement. During the use of the first exoprosthesis, two additional measurements were performed for the front limbs under similar conditions to assess the distribution of bodyweight on the forelimbs.

### Data analysis

**FIGURE 5:**
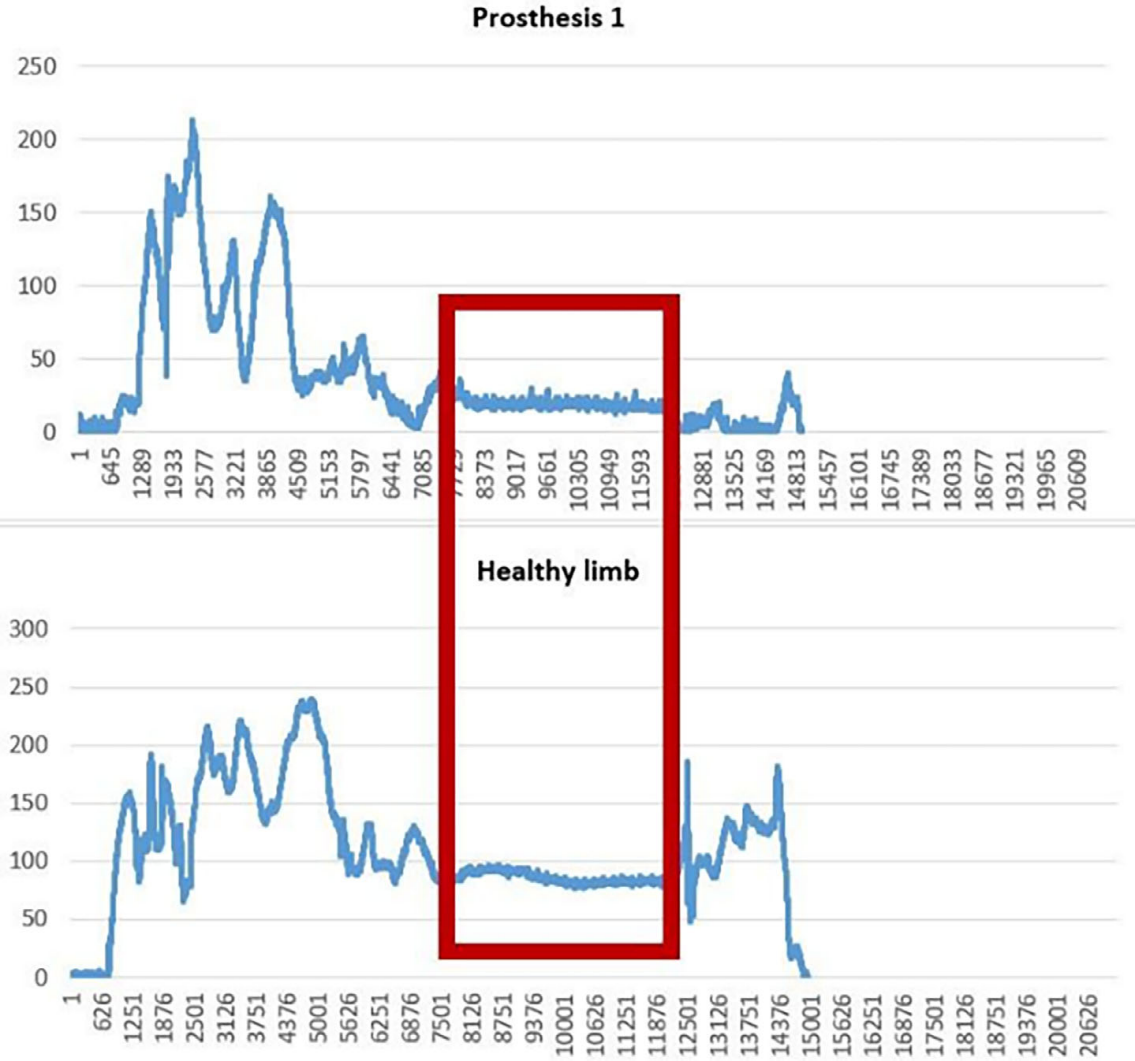
Representative example of force plate data processing. The figure illustrates a recorded force signal over time, with the segment corresponding to a stable standing phase highlighted and extracted for further analysis. Only intervals with minimal movement and consistent load distribution were considered, ensuring that the selected data accurately reflected static weight‐bearing characteristics of the limb.

Variations in measurement length occurred due to the dog's movement on the force plate. For analysis, sections where the dog stood still were isolated from the recordings. From five repetitions for each exoprosthesis, the best measurement was selected similarly, the better of the two front limb measurements was chosen. An example of the recorded measurements is shown in Figure 5. The median values for the exoprosthetic limb, the healthy hindlimb, and the front limbs were calculated from the selected data. The dog's bodyweight converted in Newtons was used to calculate GRF percentages. The weight of each prosthesis was subtracted, thereby yielding objective GRF data without the prosthesis weight influencing the GRF values.

Data from the PitPat and force plates were processed using Microsoft Excel 2016 software (Microsoft). Descriptive statistics, including the mean and standard error of the mean, were calculated.

## RESULTS

Clinical assessment revealed mild scoliosis on the right side and muscle atrophy in the right hindlimb. Range of motion showed the following results: flexion in the left hip was 52° and that of the right was 55°. After testing exoprostheses, the values were 53° and 55°. The left hip extension was 163° and the right hip extension was 165°. After testing, these values were 161° and 164°. Before exoprosthesis use the flexion in the left stifle joint was 44° and in the right was 42°. After testing, the measurements were 45° and 42°. The extension in the left stifle joint before exoprosthesis use was 166° and in the right was 161°. After testing, the values were 163° and 160°. Based on the measurements, ROM did not change during exoprosthesis testing. The dog wore each exoprosthesis for up to 5 h a day during leash walks. The first and second exoprostheses (Figure 2) were used for 7 days each. According to the owner, the dog showed reduced tolerance to the third and fourth exoprostheses, with mild skin abrasions developing on the stump after use. Testing of these exoprostheses was discontinued on the third day.

The measurements with the double force plate lasted between 2 and 165 s on the hindlimbs and between 8 and 16 s on the front limbs, depending on when the dog stepped off the plates. Measurements with inadequate positioning of the dog on the force plates were excluded.

The dog weighed 27.5 kg (269.5 N) without the exoprosthesis. Median GRF measured with force plates was 19.4 N (7.19% of body weight) with the first exoprosthesis, 18.8 N (6.97%) with the second, 12.9 N (4.78%) with the third, and 9.6 N (3.56%) with the fourth. The median GRF of the left hindlimb with the first exoprosthesis was 53.7 N (representing 19.9% of bodyweight), and with the second exoprosthesis was 57.1 N, representing 21.20% of body mass. With the third exoprosthesis, median GRF was 95.5 N (35.4% of bodyweight), and with the fourth exoprosthesis, it was 95.0 N (35.2% of bodyweight). The median GRF of the front left limb was 115.2 N, corresponding to 42.74% of bodyweight, and that of the front right limb was 77.5 N (28.75% of bodyweight). In total, the ƒorce on the two forelimbs amounted to 71.49% of the total bodyweight.

In the questionnaire, the owner reported that the dog walked more with the exoprosthesis than before using it. With all exoprostheses, the owner took the dog on one to two walks daily, covering 1.5‒3 km on varied terrain. The results of the owner‐reported questionnaire evaluating the dog's motor skills and adaptation to the exoprostheses are presented in Table 2. Overall, the second exoprosthesis demonstrated the best performance, followed by the third and first devices, while the fourth exoprosthesis achieved the lowest overall score. Representative videos illustrating the functional use of the second exoprosthesis during slow‐motion walking, running and spontaneous play in the snow are provided in Videos 1, 2, 3, respectively.

**TABLE 2:** Owner‐reported questionnaire scores evaluating motor skills and adaptation to four canine exoprostheses.

Exoprosthesis	Motor skills score (part 1) (max 25 points)	Adaptation and satisfaction score (part 2) (max 25 points)	Total score (max 50 points)	Notes
1	14	19	33	—
2	18	21	39	—
3	18	18	36	Mild stump abrasions; testing discontinued early
4	13	12	25	Mild stump abrasions; testing discontinued early

**VIDEO 1:** Slow‐motion footage of the dog walking with the second exoprosthesis. The video illustrates limb positioning and functional use of the device during controlled walking.

**VIDEO 2:** Footage of the dog running with the second exoprosthesis. The video demonstrates the use of the device during faster locomotor activity.

**VIDEO 3:** Footage of the dog playing in the snow with the second exoprosthesis. The video demonstrates the use of the device during spontaneous outdoor activity in a natural environment.

Mild skin abrasions on the stump were observed with the third and fourth exoprostheses, leading to early discontinuation of testing. These abrasions were most likely caused by improper model processing, as exoprostheses 3 and 4 were fabricated based on a different model than exoprostheses 1 and 2.

Daily walking activity monitored using the PitPat tracker and application showed variability depending on the exoprosthesis. The dog walked an average of 20.7 ± 13.4 min per day with the first exoprosthesis, which increased to 41.4 ± 14.1 min with the second exoprosthesis. Activity slightly decreased with the third exoprosthesis to 36.7 ± 15.3 min per day, while the fourth exoprosthesis showed the lowest activity levels at 20.0 ± 5.0 min per day.

**FIGURE 6:**
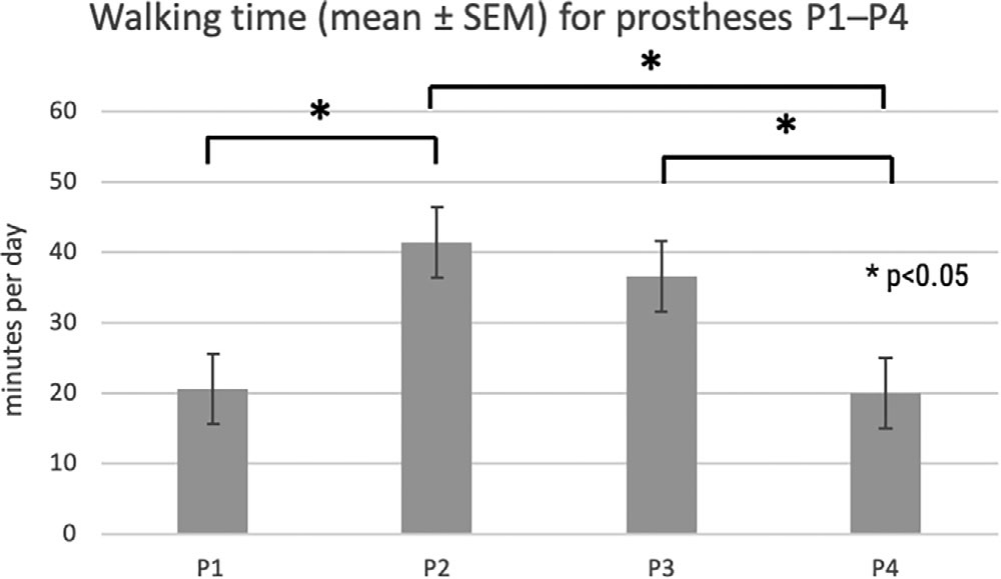
Average daily walking time (mean ± standard error of the mean [SEM]) for prostheses P1–P4.

Daily walking activity differed across exoprostheses, with the highest mean walking time observed with the second exoprosthesis and the lowest with the first and the fourth. Details are shown in Figure 6.

## DISCUSSION

The primary aim of this study was to assess the suitability of four custom‐manufactured exoprostheses for a dog using various methods, including a force plate, activity monitoring via a PitPat tracker, and owner feedback through a questionnaire.

Our analysis began by assessing the forces transmitted to different exoprosthetic designs (as well as the healthy hindlimb and front limbs) using a force plate. Additionally, we tracked the dog's daily activity using the PitPat tracker and evaluated the owner's satisfaction and observations via a questionnaire.

Range of motion measured with a goniometer before and after using the exoprosthesis showed normal values. The third and fourth exoprostheses caused slight skin abrasions on the distal medial part of the stump, which led to the discontinuation of testing for these exoprostheses. Muscles and ligaments, out of the limb's physiological position, may suffer from incorrectly distributed pressure on sensitive areas, leading to skin abrasions and blisters that could turn into larger wounds.^25^ It can therefore be concluded that the method of taking the measurement with alginate was not suitable or that errors occurred when processing the plaster model of the stump.

The force plate measurements revealed considerable load variation between the healthy and exoprosthetic limbs. This variation is likely due to the dog's movement on the plate or improper positioning. Measurements were selected where both force plates were loaded simultaneously, which provided the most consistent data (Figure 4). To improve data accuracy, we suggest incorporating video recordings to confirm the dog's position on the force plates during measurement. Additionally, dynamic measurements during walking could provide more accurate insights into the forces on the exoprosthesis than the static measurements used in this study.

Studies have shown that the normal percentage of weight transfer to a hindlimb in the standing position with equal distribution of all four limbs is 20%.^11^, ^26^ Our results indicate that the first exoprosthesis transferred 7.19% of the bodyweight to the exoprosthetic limb, which is consistent with the range of bodyweight transferred to the limb with exoprosthesis found in other studies.^6^, ^7^ The second exoprosthesis showed a similar load distribution (6.97%). The difference in load transfer between these two exoprostheses was minimal (0.22%), suggesting that the dog distributed weight similarly with both devices. Interestingly, our data also revealed a significant weight shift to the front limbs, with 71.49% of the bodyweight transferred to the forelimbs, which is higher than the 60%‒70% typically seen in healthy dogs.^10^, ^11^ Furthermore, a larger proportion of the weight was transferred to the left front limb (42.74%) compared to the right front limb (28.75%), which may be a compensatory mechanism for the amputation of the right hindlimb. Previous studies in German Shepherd dogs and French Bulldogs also noted similar compensatory shifts in weight distribution.^11^, ^26^ Increased weight transfer to the forelimbs may result in chronic overload of joints, potentially predisposing the animal to joint degeneration, pain and soft‐tissue strain over time. Furthermore, sustained compensatory loading may promote muscle imbalance and postural adaptations, which could adversely affect long‐term biomechanical function and overall musculoskeletal health.

Wendland et al.^27^ used a pressure‐sensitive gait analysis system to check the weight transfer to the exoprosthesis in 12 dogs, including two dogs with a hindlimb amputation in the metatarsal region. One dog was excluded from the study due to complications. In another dog, dynamic gait analysis during the stance phase revealed a weight shift of 16% (normal weight shift was defined by the authors as 20%). The authors found that there was a greater potential for damage to the healthy limb due to the higher loading in dogs with amputation. They also found that the paw of the remaining limbs is more elongated due to the greater mass at the phalanges and that the entire remaining limb is positioned more cranially in dogs with amputation than the limbs in dogs in the control group. In our study, we found that hindlimb amputation can also lead to certain complications, such as scoliosis, due to excessive weight transfer to the non‐amputated side of the body.

Based on the cumulative points achieved in the questionnaires for the individual exoprostheses, the results show that exoprosthesis 2 is the most suitable according to the owner's opinion and observation of the dog.

The dog consistently wore the exoprostheses during leash walks, allowing for controlled activity monitoring. The results analysed by the PitPat app revealed that the second exoprosthesis resulted in the longest walking duration and maximum activity. The third and fourth exoprostheses were discontinued after 3 days due to skin irritation. While daily variations in walking time could be influenced by factors such as weather, the consistency of the measurements minimised the impact of these fluctuations. The owner reported a significant improvement in walking ability, walking the dog up to twice a day, which had not been possible before the exoprosthesis fitting. Similar findings were observed in studies by Wendland et al.,^27^ where 72% of dog owners reported positive outcomes with exoprosthetic devices, and Pavlovic and Mursec,^12^ where 62.5% of owners were satisfied with their mobility devices.

Combining the results of the PitPat application, the owner's questionnaires, and the force plate measurements, we determined that the second exoprosthesis was the most suitable overall.

According to the results of the methods used, the first exoprosthesis had a good result on the force plate, but the dog's activity and questionnaire scores were lower than the second exoprosthesis. The third and fourth exoprostheses were unsuitable as mild skin abrasions occurred on the stump, indicating the inadequacy of the exoprosthesis. Modification of the shape of the exoprosthesis could have improved fit and eliminated skin abrasions.

This study has several limitations, including the inclusion of only a single dog, a relatively short follow‐up period for the use of the exoprostheses, no repetitions after a certain time, reliance on owner‐reported outcomes, and the absence of attempts to further optimise the fit of the third and fourth prostheses.

## CONCLUSION

This case study indicates that a multimodal assessment combining measures of limb loading and activity with owner‐reported observations may be useful for short‐term evaluation of hindlimb exoprostheses in dogs with partial limb amputation. Exoprosthesis 2, which was ultimately recommended in this study, was fabricated on the basis of stump measurements obtained using plaster bandages, 4‐mm polypropylene as the primary material and the double‐mould method for manufacturing. This exoprosthesis provided the most appropriate overall functional outcome for the dog in the present case. However, conclusions are limited by the single‐case design and potential owner feedback. Further studies involving larger cohorts, standardised protocols, longitudinal follow‐up and comprehensive kinetic gait analysis are needed to inform clinical application and optimise functional outcomes and welfare. In addition, these findings may support the broader application of animal exoprostheses in other companion animal species, such as cats, and may contribute to future research in the field of veterinary prosthetics.

## AUTHOR CONTRIBUTIONS

The study was designed and the measurements were conducted by Aljaž Muršec and Jurij Žel. The data analysis and interpretation of the results were carried out by Jurij Žel and Monika Pavlović. The manuscript was co‐written by Aljaž Muršec, Vladimira Erjavec and Monika Pavlović. The manuscript was reviewed and revised by Jurij Žel and Vladimira Erjavec. Everyone listed as an author fulfils all three of the ICMJE guidelines for authorship.

## CONFLICTS OF INTEREST

The authors declare they have no conflicts of interest.

## ETHICS STATEMENT

Ethical committee approval was not required, as the procedure involved a routine surgical intervention and subsequent rehabilitation of the animal following amputation. The animal's owner provided written informed consent for the surgical procedure, prosthetic treatment and inclusion in the study.

## Data Availability

The data that support the findings of this study are available from the corresponding author upon reasonable request.

## References

[1] Chopra T . A study on applications of prosthetic limbs in animals and use of 3D printing. BioGecko. 2023;12(2):556–60.

[2] Mendaza‐DeCal R , Peso‐Fernandez S , Rodriguez‐Quiros J . Orthotics and prosthetics by 3D‐printing: accelerating its fabrication flow. Res Vet Sci. 2023;162:104960. 10.1016/j.rvsc.2023.104960 37480718

[3] Mich PM , Kaufmann M . Veterinary orthotics and prosthetics. In: Zink MC , Van Dyke JB , editors. Canine sports medicine and rehabilitation. 2nd ed. Ames (IA): John Wiley & Sons; 2018:265–93. 10.1002/9781119380627.ch11

[4] Arauz PG , Chiriboga P , García MG , Kao I , Díaz EA . New technologies applied to canine limb prostheses: a review. Vet World. 2021;14(10):2793–802. 10.14202/vetworld.2021.2793-2802 34903941 PMC8654758

[5] Keszler MS , Heckman JT , Kaufman GE , Morgenroth DC . Advances in prosthetics and rehabilitation of individuals with limb loss. Phys Med Rehabil Clin N Am. 2019;30(2):423–37. 10.1016/j.pmr.2018.12.013 30954156

[6] Wendland TM , Seguin B , Duerr FM . Prospective evaluation of canine partial limb amputation with socket prostheses. Vet Med Sci. 2023;9(4):1521–33. 10.1002/vms3.1146 37287388 PMC10357256

[7] de Souza MMN , da Cunha Antonioli M , dos Santos MHM , dos Santos Véras BM , Carvalho LRRA . 3D exoprosthesis in socket model for dog with amputed pelvic limb: case report. BMC Vet Res. 2025;21(1):113. 10.1186/s12917-025-04574-6 40011895 PMC11863676

[8] Mendaza‐DeCal R , Peso‐Fernandez S , Rodriguez‐Quiros J . Orthotics and prosthetics by 3D‐printing: accelerating its fabrication flow. Res Vet Sci. 2023;162:104960. 10.1016/j.rvsc.2023.104960 37480718

[9] Dickerson VM , Coleman KD , Ogawa M , Saba CF , Cornell KK , Radlinsky MG , et al. Outcomes of dogs undergoing limb amputation, owner satisfaction with limb amputation procedures, and owner perceptions regarding postsurgical adaptation: 64 cases (2005‒2012). J Am Vet Med Assoc. 2015;247(7):786–92. 10.2460/javma.247.7.786 26383755

[10] Carr BJ , Canapp S , Petrovitch JL , Campana D , Canapp D , Leasure CS . Retrospective study on external canine limb prosthesis used in 24 patients. Vet Evid. 2018;3(1):1. 10.18849/ve.v3i1.118

[11] Rodriguez O , Regueiro‐Purriños M , Figueirinhas P , Gonzalo‐Orden JM , Prada I , Vilar JM , et al. Dynamic and postural changes in forelimb amputee dogs: a pilot study. Animals. 2024;14(13):13. 10.3390/ani14131960 PMC11240608 38998072

[12] Pavlović M , Mursec A . User experience of canine assistive mobility aids. Proc Socratic Lectures. 2023;8:30–7. 10.55295/PSL.2023.I5

[13] Marcellin‐Little DJ , Drum MG , Levine D , McDonald SS . Orthoses and exoprostheses for companion animals. Vet Clin North Am Small Anim Pract. 2015;45(1):167–83. 10.1016/j.cvsm.2014.09.009 25432685

[14] Carvalho LRRA . 3D printed orthopedic prostheses for domestic and wild birds—case reports. Sci Rep. 2024;14:7989. 10.1038/s41598-024-58762-9 38580783 PMC10997581

[15] Heitzmann DWW , Block J , Trinler U , Wolf SI , Alimusaj M . Motion analysis in lower limb exoprosthetics—possibilities and limitations. Orthopadie (Heidelb). 2023;52(8):631–42. 10.1007/s00132-023-04408-z 37458809

[16] Sawers A , Hafner BJ . Narrowing beam‐walking is a clinically feasible approach for assessing balance ability in lower‐limb prosthesis users. J Rehabil Med. 2018;50(5):457–64. 10.2340/16501977-2329 29616279 PMC6171346

[17] McMahon JJ , Ripley NJ , Comfort P . Force plate‐derived countermovement jump normative data and benchmarks for professional rugby league players. Sensors (Basel). 2022;22(22):8669. 10.3390/s22228669 36433265 PMC9696698

[18] Boutwell E , Stine R , Gard S . Impact testing of the residual limb: system response to changes in prosthetic stiffness. J Rehabil Res Dev. 2016;53(3):369–78. 10.1682/JRRD.2014.10.0234 27272982 PMC5555366

[19] Blumentritt S , Schmalz T , Layher F , Timmermann A , Aschoff HH . Force transmission capacity of the lower limb during walking of amputees with bone‐anchored prostheses compared with socket prostheses and persons with hip replacements. Clin Biomech (Bristol). 2023;110:106099. 10.1016/j.clinbiomech.2023.106099 37832468

[20] de Castro MP , Soares D , Mendes E , Machado L . Plantar pressures and ground reaction forces during walking of individuals with unilateral transfemoral amputation. PM R. 2014;6(8):698–707.e1. 10.1016/j.pmrj.2014.01.019 24487128

[21] Aristizabal Escobar AS , de Souza ANA , de Campos Fonseca Pinto ACB , Matera JM . Kinetic gait analysis in English Bulldogs. Acta Vet Scand. 2017;59(1):77. 10.1186/s13028-017-0344-6 29096664 PMC5669001

[22] Kyberd PJ , Poulton A . Use of accelerometers in the control of practical prosthetic arms. IEEE Trans Neural Syst Rehabil Eng. 2017;25(10):1884–91. 10.1109/TNSRE.2017.2693683 28422662

[23] Jaegger G , Marcellin‐Little DJ , Levine D . Reliability of goniometry in Labrador Retrievers. Am J Vet Res. 2002;63(7):979–86. 10.2460/ajvr.2002.63.979 12118679

[24] Choo YJ , Kim DH , Chang MC . Amputation stump management: a narrative review. World J Clin Cases. 2022;10(13):3981–8. doi: 10.12998/wjcc.v10.i13.3981 35665133 PMC9131228

[25] Portnoy S , Yarnitzky G , Yizhar Z , Kristal A , Oppenheim U , Siev‐Ner I , et al. Real‐time patient‐specific finite element analysis of internal stresses in the soft tissues of a residual limb: a new tool for prosthetic fitting. Ann Biomed Eng. 2007;35(1):120–35. 10.1007/s10439-006-9208-3 17120139

[26] Souza ANA , Pinto ACBCF , Marvulle V , Matera JM . Evaluation of vertical forces in the pads of German Shepherd dogs. Vet Comp Orthop Traumatol. 2013;26(1):6–11. 10.3415/VCOT-11-07-0100 23111688

[27] Wendland TM , Seguin B , Duerr FM . Prospective evaluation of canine partial limb amputation with socket prostheses. Vet Med Sci. 2023;9(4):1521–33. 10.1002/vms3.1146 37287388 PMC10357256

